# Should adjacent asymptomatic lumbar disc herniation of L5-S1 isthmic spondylolisthesis be simultaneously rectified? Evaluation of postoperative spino-pelvic sagittal balance and functional outcomes

**DOI:** 10.1186/s12891-022-05794-9

**Published:** 2022-09-05

**Authors:** Lei Deng, Xi Hua, Qian Wu, Nanning Lv, Xiaofeng Shao, Quan Zhou, Hao Liu, Zhonglai Qian

**Affiliations:** 1 Department of Orthopaedics, The First Affiliated Hospital of Soochow University, No.899 Pinhai Road, Suzhou, 215006 Jiangsu China; 2 Department of Orthopedic Surgery, The Second People’s Hospital of Lianyungang, 41 Hailian East Street, Lianyungang, 222003 Jiangsu China

**Keywords:** Adjacent asymptomatic lumbar disc herniation, Isthmic spondylolisthesis, Posterior lumbar interbody fusion, Sagittal balance, Reoperation

## Abstract

**Background:**

This study aimed to analyze the efficacy of the simultaneous rectification of adjacent asymptomatic lumbar disc herniation (asLDH) of L5-S1 isthmic spondylolisthesis (IS).

**Methods:**

One hundred and forty-eight patients with L5-S1 IS, and simultaneous L4-5 asLDH, were recruited between January 2012 and December 2017, for this study. Group A: seventy-two patients received PLIF at L5-S1. Group B: seventy-six patients received PLIF at L4-S1. The radiographic outcomes were assessed via the lumbar lordosis (LL), segmental lordosis (SL), sacral slope (SS), pelvic incidence (PI), pelvic tilt (PT), PI-LL and slip degree (SD). The functional outcomes were evaluated via the visual analog scale (VAS), Oswestry disability index (ODI), and reoperation rate. The potential risk hazards for reoperation were identified using both uni- and multivariate logistic regression analyses.

**Results:**

The postoperative LL, SL, PT, SS, SD, VAS, and ODI exhibited vast improvements (*P* < 0.05). Relative to Group A, Group B exhibited markedly better LL, SL, PT, PI-LL,VAS and ODI scores at the final follow-up (*P* < 0.05). Group B also achieved better SD values post surgery than Group A (*P* < 0.05). The reoperation rate was remarkably elevated in Group A, compared to Group B (*P* < 0.05). The multivariate logistic regression analysis showed the L4-5 asLDH grade was a stand-alone risk hazard for reoperation, whereas, pre-SL and pre-LL offered protection against reoperation (*P* < 0.05).

**Conclusions:**

L4-S1 PLIF is recommended to correct asLDH in L5-S1 IS patients, with high-grade disc herniation and abnormal sagittal alignment.

## Introduction

Isthmic spondylolisthesis (IS) is a common disorder involving spinal surgery, and it is characterized by the partial or total spondylolisthesis of the upper and lower vertebrae caused by isthmus disconnection. The L5-S1 level is commonly affected by IS, and accounts for about 71–95% of all patients with IS [1, 2]. The most common L5-S1 IS symptoms include lower back pain, lumbar instability, and pain in one or both legs, caused by L5 nerve root compression [3, 4]. Surgical intervention can successfully relieve nerve compression and low back pain, restore intervertebral space height, and correct lumbar spondylolisthesis deformity [5]. Posterior lumbar interbody fusion (PLIF) is a classical surgery that treats IS. Multiple researches described remarkable clinical outcomes in PLIF-based IS treatment [6–8].

To treat L5-S1 IS, PLIF is generally performed at the L5-S1 level. However, several studies demonstrated that the sagittal spine balance recovers poorly following single-level PLIF surgery [9]. The lumbar spine sagittal imbalance typically leads to adjacent segment degeneration (ASD) [10, 11]. Simultaneously, multiple reports suggested that PLIF is another risk factor for ASD [12–14]. Clinically, multiple patients with L5-S1 IS also experienced L4-5 adjacent asymptomatic lumbar disc herniation (asLDH). Yeon Heo et al. suggested that patients who undergo L5-S1 fusion surgery for IS are at a higher risk of developing clinical symptomatic ASD post operation because the synergistic effects of discectomy, fusion, and fixation can accelerate lumbar disc degeneration [15]. Moreover, in patients, who do not exhibit signs and symptoms related to L4-5 disc herniation prior to surgery, a pre-existing herniation may likely alter an asymptomatic LDH into a symptomatic one, which would require a secondary surgery. Striking the right balance between reduced reoperation rates and minimal surgical trauma is a challenge for most physicians. In addition, lack of proper data makes it difficult to determine the risks and benefits of including asLDH in the primary surgery.

Hence, the purpose of this study was to assess whether asLDH of L5-S1 IS should be simultaneously rectified, along with PLIF surgery, and the primary endpoints were spino-pelvic sagittal balance and functional outcomes.

## Methods

### Selection criteria

The inclusion criteria were as follows: (1) X-ray and CT imaging-based diagnosis of L5-S1 IS (X-ray and CT showed L5 vertebral spondylolisthesis forward, L5 vertebral isthmus bone discontinuous), and confirmed indication for surgery (including persistent symptoms after nonsurgical or interventional treatment, and significant or progressive neurological deficits). (2) MRI-confirmed L4-5 disc herniation (MRI showed contact or compression between the L4-5 disc material and nerve root or the dura mater), but without the symptoms of nerve compression and physical examination related to the L4-5 herniated disc. (3) The asLDH severity evaluated as ≥ Grade 1, based on a report by Pfirrmann et al. [16]. (4) PLIF conducted at L5-S1 or L4-S1. The screws, rods, and cages employed in PLIF were produced by the same company. (5) The follow-up period lasted a minimum of three years, and all relevant information was present.

The exclusion criteria were as follows: (1) Patients with other lumber disc herniation, except for L4-S1 disc herniation. (2) Patients with a history of spinal surgery or fractures. (3) Patients with intervertebral space infections, spinal tumor or tuberculosis, and congenital spinal deformities.

### Patient demographics

In line with the aforementioned inclusion and exclusion criteria, one hundred and forty-eight patients with L5-S1 IS and L4-5 asLDH were recruited between January 2012 and December 2017 for our retrospective analysis. The patients were separated into Groups A and B. Group A (*n* = 72) received PLIF at L5-S1 level, and Group B (*n* = 76) received PLIF at the L4-S1 level. The medical ethics committee approved the informed consent forms signed by all participants and their families. We analyzed the postsurgical sagittal balance and functional outcomes between Groups A and B. To examine the hazard risk for reoperation, Group A was further separated into two subpopulations, based on reoperation after the final follow-up. Twenty-five patients were grouped into Group A1, which represented patients who underwent reoperation after the last follow-up, and forty-seven patients comprised Group A2, which represented no reoperation after the final follow-up. Univariate analysis assessed significant differences between the two subpopulations. Subsequently, meaningful indicators were entered into binary multivariate analysis to identify stand-alone hazard risk(s) for reoperation.

### Surgical technique

To minimize variability between surgeries, all operations were carried out by the same two experienced orthopedic surgeons. All patients received general anesthesia and were placed prone to initiate the operation. Using C-arm fluoroscopy, the affected segment entry point was identified. Next, an incision was made in the midline to expose the spinous processes, laminae, and transverse processes.

**Fig. 1:**
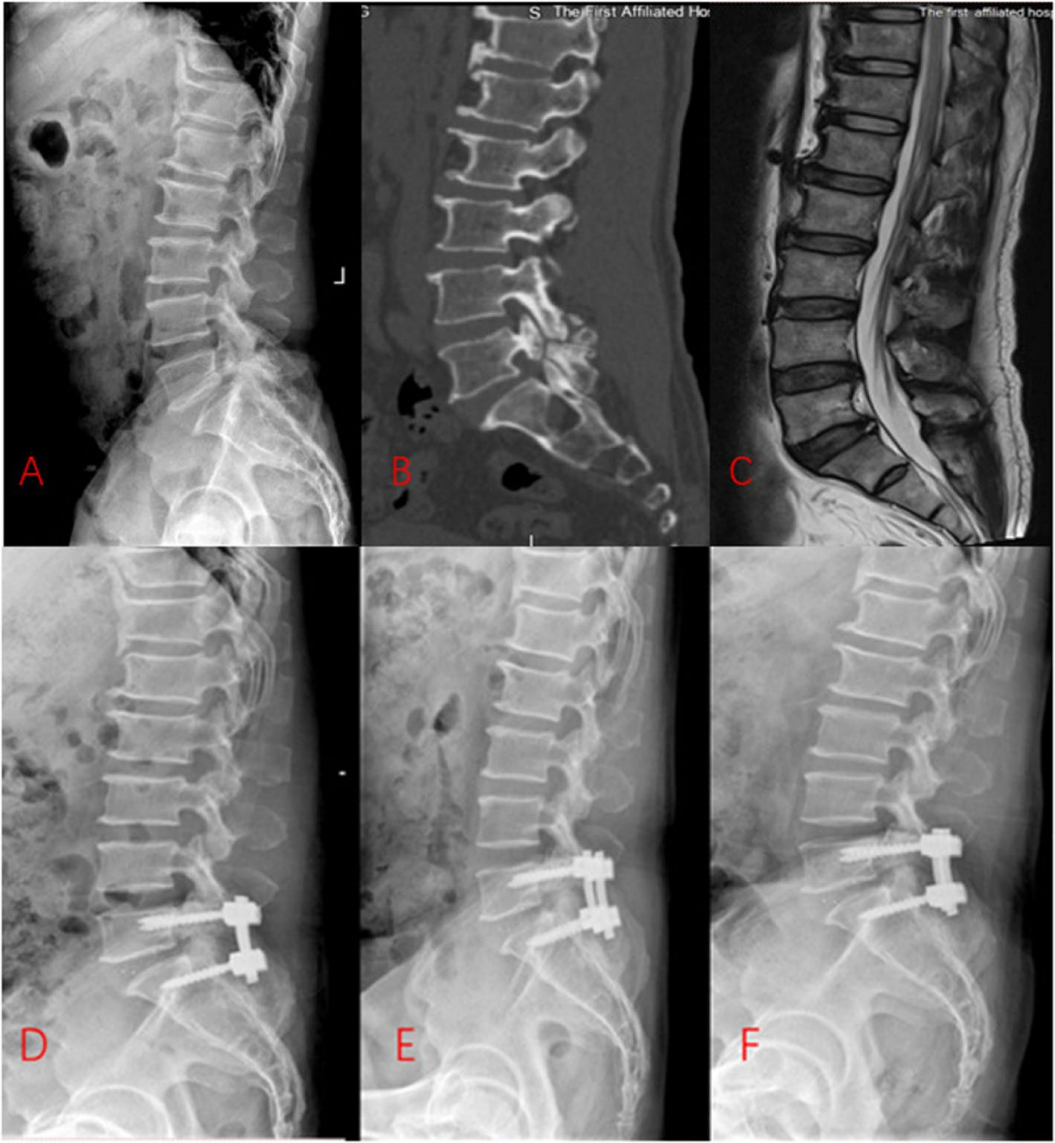
The preoperative and postoperative radiographs of Group A. **A**, Preoperative lateral X-ray. **B**, Preoperative computed tomographic scan. **C**, Preoperative T2-weighted magnetic resonance image. **D**, Lateral X-ray 1 months after surgery. **E**, Lateral X-ray 1 year after surgery. **F**, Lateral X-ray at final follow-up

Group A: Two pedicle screws were routinely implanted in each of the L5 and S1 vertebrae under C-arm X-ray fluoroscopic guidance. Overall, 4 pedicle screws were placed. Then, the L5 and S1 vertebral bilateral ligamentum flavum and lamina were removed to expose the L5-S1 disc. An incision was made into the annulus fibrosus and disc, and the disc tissue was removed using a ring curette. Following a complete discectomy, a cage of appropriate size was placed between the L5-S1 vertebrae. Immediately after the lifting and reduction were completed, titanium rods were inserted, and the nut was locked in place (Fig. 1).

**Fig. 2:**
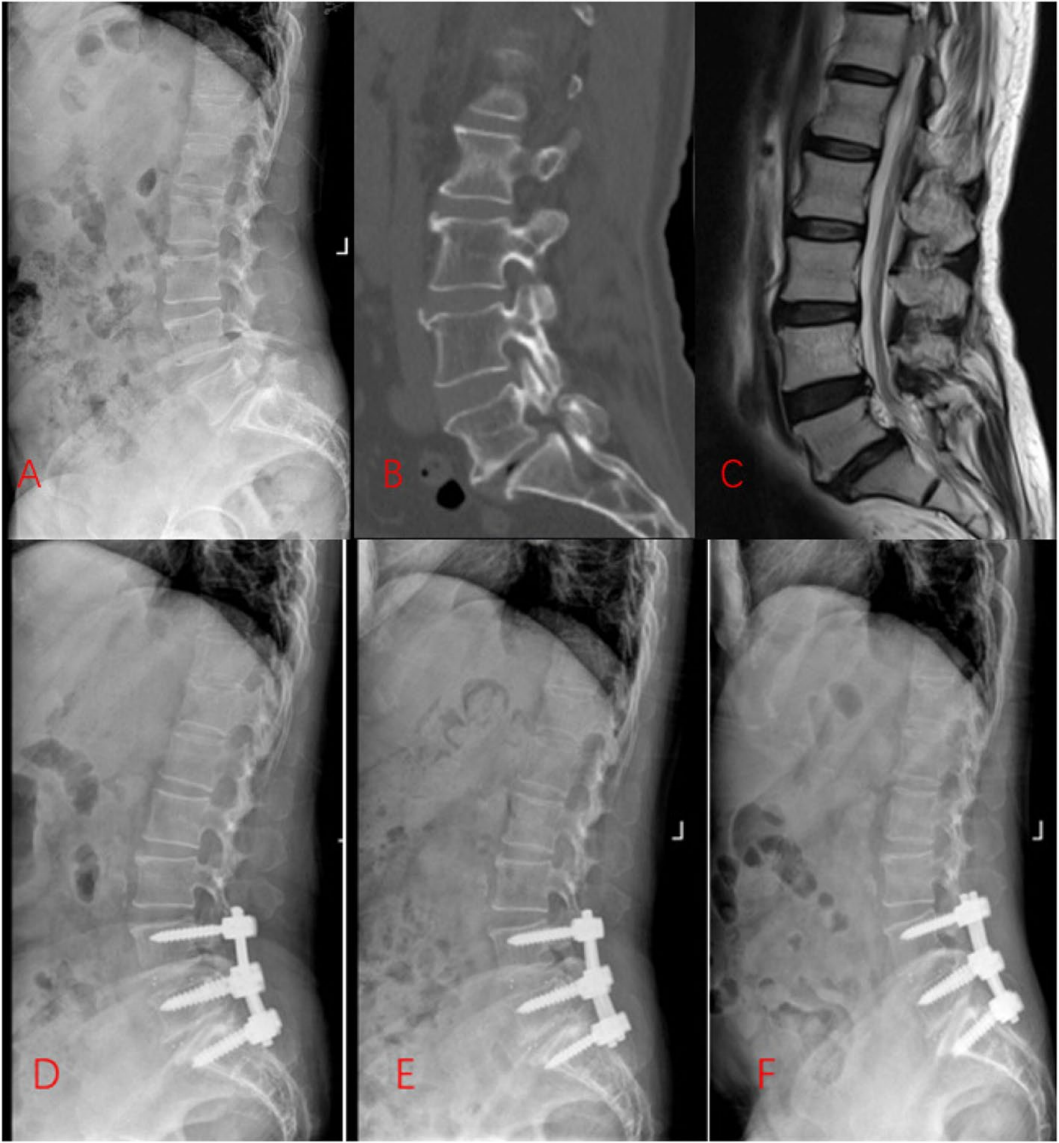
The preoperative and postoperative radiographs of Group B. **A**, Preoperative lateral X-ray. **B**, Preoperative computed tomographic scan. **C**, Preoperative T2-weighted magnetic resonance image. **D**, Lateral X-ray 1 months after surgery. **E**, Lateral X-ray 1 year after surgery. **F**, Lateral X-ray at final follow-up

Group B: Two pedicle screws were routinely implanted in each of the L4, L5, and S1 vertebrae under C-arm X-ray fluoroscopic guidance. The L4, L5, and S1 vertebral bilateral ligamentum flavum and lamina were then removed to expose the L4-L5 and L5-S1 discs. An incision was made into the annulus fibrosus and disc, and the disc tissue was excised using a ring curette. Following a complete disectomy, two cages of appropriate sizes were respectively placed between the L4-L5 vertebrae and between the L5-S1 vertebrae. Immediately after the lifting and reduction were completed, titanium rods were inserted, and the nut was locked in place (Fig. 2).

### Assessed parameters

#### Clinical assessment

The visual analogue scale (VAS) was employed to determine the patient’s perception of lower back pain prior to surgery, as well as 1 month, 1 year, and at the final follow-up after surgery (0–10 scale, with 0 being painless and 10 being the most painful) [17].Moreover, the Oswestry disability index (ODI) was employed for quality of life assessment prior to surgery, as well as 1 month, 1 year, and at the final follow-up after surgery [18]. The reoperation rate was employed to assess incidences of patients undergoing PLIF reoperation for symptoms and signs related to the L4-5 disc herniation by the final follow-up.

#### Radiographic evaluation

**Fig. 3:**
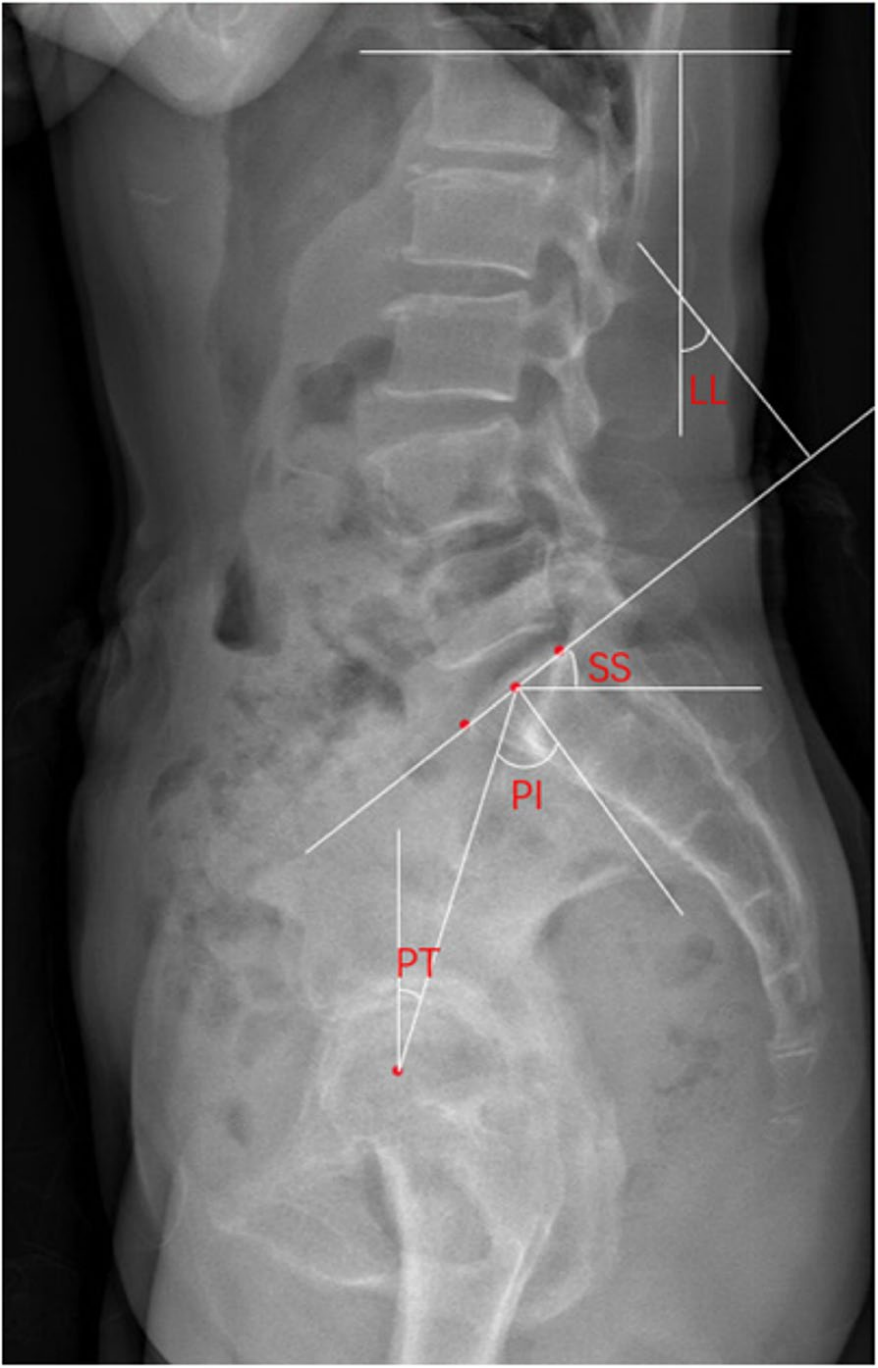
Plain lateral radiographs for measuring spino-pelvic sagittal parameters. LL: Lumbar lordosis, SS: Sacral slope, PI: Pelvic incidence, PT: Pelvic tilt

**Fig. 4:**
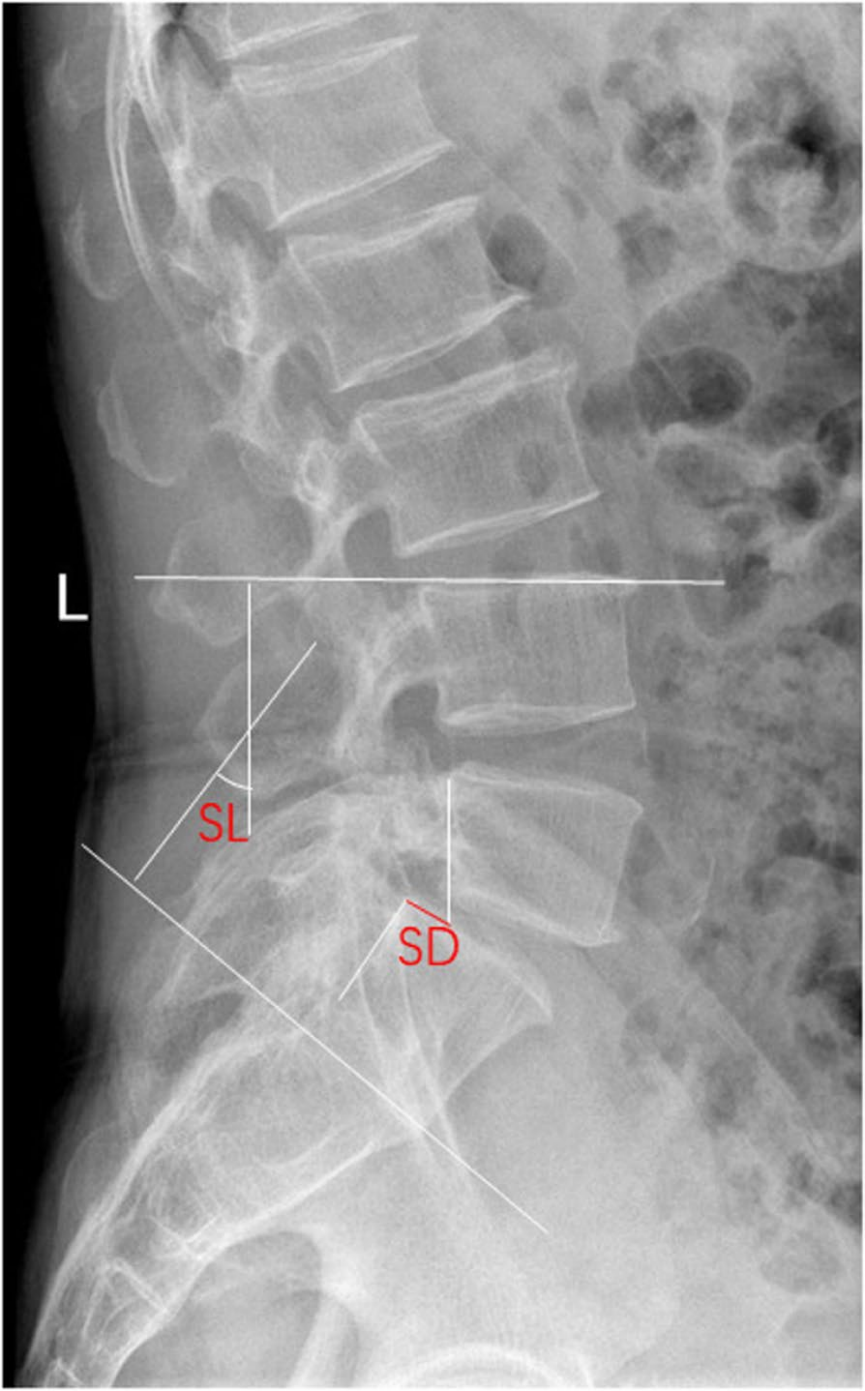
Plain lateral radiographs for measuring local parameters. SD: Slip degree, SL: Segment lordosis

All patients underwent anteroposterior and lateral radiograph imaging prior to surgery, as well as 1 month, 1 year, and at the final follow-up after surgery. The radiological recordings were conducted by three experienced spinal surgeons, and assessment was carried out via the blinding method. Per patient, the three independent radiological recordings showed a difference of less than 5%, thus, suggesting accurate, stable, and reliable measurements. The mean of each radiographic parameter was used during analysis. The radiographic variables were measured as follows: Segmental lordosis (SL), the angle between the lower endplates of the upper vertebrae and the lower endplates of the responsible vertebrae. Lumbar lordosis (LL), the angle between the upper endplate of the L1 vertebra and the sacral plate. The sacral slope (SS), the angle between the sacral plate and the horizontal line. The pelvic incidence (PI), the angle between the line perpendicular to the midpoint of the sacral plate and the line connecting the femoral head midpoint to the sacral plate midpoint. The pelvic tilt (PT), the angle made by a vertical line of the sacral plate midpoint and the femoral head axis. Slip degree (SD), determined by the Meyerding grade (Fig. 3 and Fig. 4).

#### Statistical methods

All data analyses employed the SPSS 26.0 software, and data are presented as the mean ± standard deviation. Inter-group variable comparisons were carried out via the paired sample and independent-simple t tests. Categorical data were assessed via the χ^2^ and Fisher exact tests. Univariate analysis compared between the two groups. Significant indicators from the univariate analysis were entered into multivariate analysis to identify stand-alone reoperation risk factors. *P* < 0.05 was set as the significance threshold.

## Results

### Demographics

Table 1 summarizes Group A and Group B patient information. According to PLIF involving different segments, seventy-two patients were grouped into Group A (L5-S1 PLIF) with 28 male patients and 44 female patients. In addition, seventy-six patients were grouped into Group B (L4-S1 PLIF), with 34 male patients and 42 female patients. All participants completed a minimum of 36 months of follow-up, with Group A completing 57.15 ± 10.12 months, and Group B 59.17 ± 9.65 months. No obvious differences were evident in patient age, gender, body mass index (BMI), subcutaneous fat index (SFI), bone mineral density (BMD), meyerding grade, L4-5 asLDH grade, and follow-up times of patients in Groups A and B (*P* > 0.05).

**Table 1:** The demographic data of groups

	Group A(*N* = 72)	Group B(*N* = 76)	*P*-value
Age (years)	53.82 ± 8.62	55.51 ± 9.66	0.263
Gender(male/female)	28/54	34/52	0.469
BMI(kg/m^2^)	24.50 ± 2.97	25.27 ± 2.74	0.104
SFI (mm)	8.54 ± 2.94	9.20 ± 2.69	0.152
BMD(T-score)	-1.82 ± 0.27	-1.84 ± 0.28	0.585
Meyerding grade			
I	24	22	0.564
II	48	22	
L4-5 asLDH grade			
Grade 1	22	24	0.893
Grade 2	50	52	
Follow-up(months)	57.15 ± 10.12	59.17 ± 9.65	0.214

*BMI* Body mass index, *SFI* Subcutaneous fat index, *BMD* Bone mineral density, *asLDH* adjacent asymptomatic lumbar disc herniation

### Evaluation of intra-rater and inter-rater reliability

Table 2 lists inter-observer and intra-observer intraclass correlation coefficient (ICC) values. The ICC values showed excellent agreement (> 0.9) for all measurements. None of the differences were statistically significant.

**Table 2:** Inter-observer and intra-observer intraclass correlation coefficients for all sagittal alignment parameters

	Inter-observer intraclass correlation coefficients (ICC)				Intra-observer intraclass correlation coefficients (ICC)			
	Pre (95%CI)	1 months (95%CI)	1 year (95%CI)	Final (95%CI)	Pre (95%CI)	1 months (95%CI)	1 year (95%CI)	Final (95%CI)
SL	0.98(0.97,0.99)	1.00(1.00,1.00)	0.99(0.99,1.00)	1.00(1.00,1.00)	0.95(0.92,0.97)	1.00(1.00,1.00)	1.00(1.00,1.00)	1.00(1.00,1.00)
LL	0.98(0.98,0.99)	1.00(1.00,1.00)	0.99(0.99,0.99)	0.99(0.98,0.99)	0.95(0.93,0.97)	0.99(0.99,0.99)	0.97(0.96,0.98)	0.96(0.95,0.97)
PI	1.00(1.00,1.00)	1.00(1.00,1.00)	1.00(1.00,1.00)	1.00(1.00,1.00)	1.00(1.00,1.00)	1.00(1.00,1.00)	1.00(1.00,1.00)	1.00(1.00,1.00)
PT	1.00(1.00,1.00)	0.99(0.98,0.99)	1.00(1.00,1.00)	0.98(0.97,0.99)	0.99(0.99,1.00)	0.97(0.96,0.98)	1.00(1.00,1.00)	0.96(0.93,0.97)
SS	0.94(0.93,0.96)	0.99(0.99,1.00)	0.97(0.95,0.99)	1.00(1.00,1.00)	0.95(0.94,0.97)	0.98(0.97,0.99)	0.98(0.98,0.99)	1.00(1.00,1.00)
SD	1.00(1.00,1.00)	1.00(1.00,1.00)	1.00(1.00,1.00)	1.00(1.00,1.00)	1.00(1.00,1.00)	1.00(1.00,1.00)	1.00(1.00,1.00)	1.00(1.00,1.00)

*SL* Segmental lordosis, *LL* Lumbar lordosis, *SS* Sacral slope, *PT* Pelvic tilt, *PI* Pelvic incidence, *SD* Slip degree, *Pre* preoperative

### Radiographic prognosis

Table 3 lists the radiographic prognoses of Groups A and B. The LL, SL, PT, SS, PI-LL and SD post operation demonstrated great improvements, relative to the corresponding preoperative values in both groups (*P* < 0.05). Relative to Group A, Group B exhibited markedly better LL, SL PI-LL and PT outcomes at the final follow-up (*P* < 0.05). Likewise, the postoperative SD was better in Group B versus A (*P* < 0.05). No discernible differences were observed in other parameters between the two groups.

**Table 3:** The radiographic data of groups

	Group A(*N* = 72)	Group B(*N* = 76)	*P*-value
SL (°)			
Pre	22.53 ± 2.47	23.25 ± 3.10	0.121
1 months	27.58 ± 3.50*	28.26 ± 5.17*	0.353
1 year	27.49 ± 2.86*	28.51 ± 4.54*	0.104
Final	26.79 ± 3.44*	28.13 ± 3.92*	**0.029**
LL (°)			
Pre	51.47 ± 5.13	50.68 ± 3.53	0.276
1 months	56.69 ± 3.26*	57.54 ± 3.60*	0.137
1 year	56.76 ± 3.40*	57.50 ± 3.37*	0.189
Final	54.97 ± 3.28*	56.64 ± 3.70*	**0.004**
PI (°)			
Pre	61.42 ± 5.79	62.17 ± 4.81	0.389
1 months	62.65 ± 3.67	62.67 ± 4.25	0.866
1 year	62.61 ± 4.55	62.53 ± 3.67	0.901
Final	62.14 ± 4.42	61.37 ± 4.49	0.295
PT (°)			
Pre	20.74 ± 3.82	20.61 ± 4.06	0.840
1 months	16.74 ± 3.23*	16.05 ± 2.37*	0.142
1 year	17.01 ± 2.78*	16.24 ± 3.20*	0.118
Final	17.53 ± 2.64*	16.17 ± 2.52*	**0.002**
SS (°)			
Pre	40.68 ± 4.32	41.57 ± 3.81	0.188
1 months	45.92 ± 3.55*	46.71 ± 3.40*	0.167
1 year	45.60 ± 3.57*	46.29 ± 3.48*	0.234
Final	44.61 ± 3.57*	45.20 ± 3.55*	0.318
PI-LL (°)			
Pre	9.94 ± 7.21	11.49 ± 6.65	0.178
1 months	5.96 ± 4.94*	5.22 ± 5.28*	0.384
1 year	5.85 ± 6.04*	5.03 ± 4.84*	0.362
Final	7.17 ± 5.90*	4.72 ± 5.86*	**0.013**
SD (%)			
Pre	23.28 ± 3.45	22.43 ± 3.34	0.133
1 months	6.42 ± 1.86*	5.46 ± 1.74*	**0.001**
1 year	7.03 ± 2.13*	5.76 ± 2.01*	** < 0.001**
Final	8.44 ± 2.29*	6.14 ± 2.12*	** < 0.001**

*SL* Segmental lordosis, *LL* Lumbar lordosis, *SS* Sacral slope, *PT* Pelvic tilt, *PI* Pelvic incidence, *SD* Slip degree, *Pre* Preoperative

^*^Statistically significant compared with the preoperative, *p* < 0.05

Blod represents there is statistical significance between the groups

### Functional prognoses

Table 4 summarizes the functional prognoses of Groups A and B. The postoperative VAS and ODI were markedly different, relative to the corresponding preoperative values in both groups (*P* < 0.05). Moreover, the final follow-up VAS and ODI showed considerably elevated scores in Group A versus B (*P* < 0.05). In fact, twenty-five Group A patients experienced such severe L4-5 LDH-related pain symptoms at the final follow-up that they were indicated for reoperation. Hence, the Group A reoperation rate was 34.72%. Interestingly, no patients from Group B underwent reoperation. Therefore, the Group A reoperation rate was markedly elevated, compared to Group B (*P* < 0.05).

**Table 4:** The functional outcomes of groups

	Group A(*N* = 72)	Group B(*N* = 76)	*P*-value
VAS			
Pre	7.46 ± 0.95	7.30 ± 0.80	0.281
1 months	3.26 ± 0.73*	3.45 ± 0.77*	0.141
1 year	2.39 ± 0.57*	2.37 ± 0.54*	0.823
Final	3.74 ± 0.99*	2.66 ± 0.66*	** < 0.001**
ODI			
Pre	53.78 ± 7.18	52.58 ± 6.73	0.296
1 months	24.29 ± 3.15*	25.33 ± 3.93*	0.079
1 year	19.94 ± 3.09*	19.39 ± 3.26*	0.295
Final	31.94 ± 3.09*	19.91 ± 3.26*	** < 0.001**
Reoperation rate	34.72% (25/72)	0%	** < 0.001**

*VAS* Visual analogue scale, *ODI* Oswestry disability index, *Pre* Preoperative

^*^Statistically significant compared with the preoperative, *p* < 0.05

Blod represents there is statistical significance between the groups

### Univariate logistic regression analysis of risk factors

Table 5 shows the univariate logistic regression analysis of risk factors. The result shows significant differences in L4-5 asLDH grade (OR = 0.117, *P* < 0.05, 95%CI = 0.025–0.557), pre-SL (OR = 0.624, *P* < 0.05, 95%CI = 0.463–0.841), pre-LL (OR = 0.791, *P* < 0.05, 95%CI = 0.681–0.920), pre-ODI (OR = 1.148, *P* < 0.05, 95%CI = 0.995–1.148).

**Table 5:** Univariate logistic regression analysis for reoperation after surgery

Factor	B-value	Odds Ratio (95% Confidence interval)	*P*-value
Age	0.014	1.014(0.958–1.074)	0.634
SFI	0.124	1.132(0.953–1.344)	0.159
L4-5 asLDH grade	-2.142	0.117(0.025–0.557)	**0.007**
Pre-SL	-0.471	0.624(0.463–0.841)	**0.002**
Pre-LL	-0.234	0.791(0.681–0.920)	**0.002**
Pre-PI	0.038	1.039(0.954–1.131)	0.378
Pre-PT	0.020	1.020(0.897–1.159)	0.764
Pre-SS	0.054	1.055(0.941–1.184)	0.358
Pre-VAS	-0.032	0.969(0.579–1.621)	0.904
Pre-ODI	0.067	1.069(0.995–1.148)	**0.068**

*SFI* Subcutaneous fat index, *asLDH* Adjacent asymptomatic lumbar disc, *SL* Segmental lordosis, *LL* Lumbar lordosis, *PT* Pelvic tilt, *PI* Pelvic incidence, *SS* Sacral slope, *VAS* Visual analogue scale, *ODI* Oswestry disability index, *Pre* preoperative

Blod represents there is statistical significance

### Multivariate logistic regression analysis of risk factors

Table 6 reveals the results of multivariate analyses, with reoperation as the dependent, and SFI, L4-5 asLDH grade, pre-SL, pre-LL, and pre-ODI as the independent variable. The results show that L4-5 asLDH grade (OR = 0.124, *P* < 0.05, 95%CI = 0.022–0.708) was a strong stand-alone indicator of reoperation, whereas pre-SL (OR = 0.605, *P* < 0.05,95% CI = 0.605–0.414) and pre-LL (OR = 0.830, *P* < 0.05, 95%CI = 0.700–0.984) were protective factors for reoperation.

**Table 6:** Multivariate Logistic regression analysis for reoperation after surgery

Factor	B-value	Odds Ratio (95% Confidence interval)	*P*-value
SFI	0.035	1.035(0.820–1.307)	0.770
L4-5 asLDH grade	-2.090	0.124(0.022–0.708)	**0.019**
Pre-SL	-0.502	0.605(0.414–0.884)	**0.009**
Pre-LL	-0.187	0.830(0.700–0.984)	**0.032**
Pre-ODI	0.009	1.009(0.904–1.125)	0.878

*SFI* Subcutaneous fat index, *asLDH* Adjacent asymptomatic lumbar disc, *SL* Segmental lordosis, *LL* Lumbar lordosis, *ODI* Oswestry disability index

Blod represents there is statistical significance

## Discussion

Isthmic spondylolisthesis, brought on by a pars interarticularis fracture, involves the forward movement of one vertebral body relative to adjacent vertebral bodies. Initial treatment includes oral anti-inflammatory drugs and physical therapy. If radiculopathy is predominant, transforaminal epidural injection of corticosteroids may provide temporary relief [19]. It has been reported that bilateral transforaminal epidural steroid injections provided 54.39 ± 34.31% pain relief in patients with IS [20]. Although it has also been reported that only 31.3% of patients with IS chose to have surgery in 3-years follow-up after comprehensive nonsurgical treatment, surgery is indicated for patients with persistent symptoms despite nonoperative or interventional injection treatments, and for patients with significant or progressive neurologic deficits [19, 21]. There are also many studies that show that surgery tends to yield better patient-reported health-related outcomes compared with nonoperative management for patients with lumbar isthmic spondylolisthesis[22–24]. Owing to the instability and stress alteration of the sliding segment, the stress and shear force on the upper disc becomes enhanced, thus, resulting in ASD. In the meantime, studies revealed that fusion surgery, particularly, those in combination with laminectomy, accelerate adjacent disc degeneration [25]. This may be due to the increased stiffness of the fusion segment, which results in a rise in compensatory motion of the adjacent mobile segment, which, in turn, produces an enhanced load on the posterior facet joint [26–28]. Motion segment instabilities result from a rupture of the posterior ligamentous complex following laminectomy, which, in turn, accelerates adjacent disc degeneration [29, 30]. A study by Wu et al. revealed that when encountering asLDH, there is a high possibility of reoperation post surgery, regardless of the type of surgery (for example, open fusion or minimally invasive non-fusion) [31]. Surgeons often face a challenge regarding L5-S1 IS associated with the L4-5 asLDH. On one hand, if asLDH is left untreated, there is a high probability that symptoms may appear shortly after surgery, thus requiring a reoperation. On the other hand, double-level surgery poses greater risks, costs, and postoperative complications. This investigation retrospectively analyzed the sagittal balance and functional outcomes following both L4-S1 and L5-S1 PLIF surgeries.

It is generally accepted that the sagittal positioning of the spine is critical for lumbar degeneration to ensue [32]. In this study, the postoperative LL, SL, PT, SS, and SD demonstrated far better values, relative to the corresponding preoperative values in both groups. This indicated that post PLIF, IS patients achieved better reduction of the slipped vertebra and better recovery of the sagittal balance of the entire spine. Moreover, reduction of lumbar spondylolisthesis fully restored the spinal canal volume, relieved nerve root compression, and improved vertebral body sequence [9, 33].We observed no obvious differences in the sagittal balance parameters at 1 month and 1 year post surgery between the two groups. However, the final follow-up LL, SL, and PT in Group B was considerably better than Group A. This indicated that the L4-S1 PLIF surgery was better at maintaining long-term sagittal balance in the patient spine. Kim et al. suggested that reduced postoperative segmental lordosis angle (particularly, < 20) was strongly associated with postoperative adjacent degeneration in spondylolisthesis patients [12]. Keller et al. reported that the LL at the final follow-up is the most important risk factor for ASD [34]. Bae et al. speculated that SL is significantly correlated with adjacent segment degeneration. Thus, restoration of normal SL is crucial to preventing adjacent segment degeneration [35]. We hypothesized that the L4-S1 PLIF surgery for simultaneous treatment of asLDH can produce stronger lifting force, evenly distributed stress, and augmented safety reduction via six pedicle screw lifting reduction and two-level fusion fixation. Thus, it is better restoring the injured vertebral segment lordosis angle, as well as the physiological lumbar lordosis angle. This may also explain the vastly reduced Group B postoperative SD value versus Group A. The L4-S1 level PLIF surgery completely fuses the slipped vertebra with the upper and lower vertebrae, thus restoring the slipped vertebrae as much as possible, while avoiding re-slippage between the slipped vertebrae and the upper vertebrae in the long-term postoperative life.

The essential pelvic parameters are PI, PT, and SS. SS is described as the angle between the horizontal and parallel sacral plates S1, and it is roughly 41° ± 8°. PT represents pelvic rotation, which reduces with anteversion and enhances with retroversion [36], and it has a standard value of 13° ± 6° [37]. In Groups A and B, the mean presurgical PT slightly exceeded the upper limit, and was restored to normal at 1 year, and at the final follow-up after surgery. Multiple reports suggested a strong correlation between PT and good clinical outcomes [38, 39]. This could also explain the significant decrease in postoperative ODI and VAS scores in both groups. A normal PI value is about 53° ± 9°, and it determines pelvic positioning. All other pelvic variables (PT and SS), along with the spinal curvature, were adjusted accordingly. The aforementioned three pelvic variables were next entered into the following equation: PI = PT + SS [40]. Recently, a new parameter, PI-LL, has been produced to directly quantify the mismatch between pelvis shape and lumbar curve. There is a close relationship between LL and PI. In general, the extent of LL depends on the value of PI, and the ideal formula is: LL = PI ± 9. If these two parameters do not match, it will cause the imbalance of sagittal balance of lumbar spine. We found significant improvement in postoperative PI-LL in both groups. We observed no discernible differences in the PI and SS values between Groups A and B. However, PT and PI-LL showed marked differences between Groups A and B at the last follow-up. We, thus, speculated that the fixation strength and stress distribution were far better after double level fixation and fusion than after single level fixation.

In terms of the functional outcomes, we observed marked decreases in postoperative ODI and VAS scores in Groups A and B. No discernible differences were observed in the VAS and ODI scores at 1 month and 1 year post operation. However, these scores were considerably elevated at the final follow-up in Group A versus B. As a result, Group A had substantially elevated reoperation rate than Group B. This was primarily because the L4-5 asLDH was not surgically intervened during the initial operation, and the postoperative back pain and lower limb numbness, caused by L4-5 LDH compression of nerve roots, negatively impacted patient quality of life, and eventually required a repeat surgery. Kepler et al. speculated that a reduced postoperative SL indicates more obvious pain in the lower back and legs, as well as a higher VAS score [41]. In our study, among the 72 patients, who did not undergo surgical intervention on L4-5 asLDH, 25 patients underwent reoperation due to the deterioration of L4-5 asLDH, and the reoperation rate was 34.72%. We next attempted to predict the risk factors governing reoperation using logistic regression analysis. Our results revealed that the preoperative L4-5 asLDH grade and preoperative SL and LL were essential factors in predicting postoperative reoperation. A study by Wu et al. revealed that the reoperation rate of Grade 2 asLDH patients post fusion was considerably higher than Grade 1 patients [31]. Bae et al. speculated that the preoperative SL, LL, and postoperative SL are critical factors regulating ASD risk. Patients with preoperative sagittal abnormalities may also be prone to ASD [35]. Our investigation revealed that patients with higher preoperative asLDH grade may be more susceptible to reoperation following surgery. Moreover, preoperative SL and LL were critical factors in predicting reoperation risk. We, therefore, speculated that patients with abnormal preoperative sagittal alignment of SL and LL were more likely develop spinal instability following L5-S1 PLIF surgery. Long-term sagittal spine imbalance can result in the degeneration and aggravation of adjacent segments, and these patients are more likely to experience postoperative symptoms and dysfunction that necessitate reoperation. This will not only impact patients' long-term quality of life following surgery, but also enhance risk of secondary trauma and anesthesia to patients, particularly, elderly patients. A recent study showed that subcutaneous fat index is superior to BMI in predicting spinal degeneration with valuable cut off for both genders [42]. Our regression analysis result showed that preoperative SFI was not an independent risk factor or protective factor for reoperation. This may be due to the limitations of our study, such as small sample size and insufficient follow-up time, so further and more in-depth studies on SFI reoperation prediction are needed in the future. Therefore, in case of asLDH of L5-S1 IS patients, with high-grade disc herniation and abnormal sagittal alignment, L4-S1 PLIF is recommended during the primary surgery to avoid reoperation. Indeed, compared to L5-S1 PLIF, L4-S1 also includes certain defects that cannot be ignored. For example, longer operation time and more intraoperative bleeding can enhance patient trauma. In addition, this brings about a greater economic burden. We, therefore, need to communicate this information to the patients and their families extensively prior to surgery.

Our research encountered certain limitations. First, our patient population was relatively small (148 patients). A larger patient population is needed to obtain more meaningful statistical data. Second, there is a necessity to perform future prospective randomized controlled trials to validate our results. Finally, certain pre- and postoperative lumbar radiographs did not include the bilateral femoral head. Hence, we could only estimate the central position of the femoral head by observing the acetabular shape, which can result in measurement errors in pelvic parameters.

## Conclusions

In case of L4-5 asLDH of L5-S1 IS patients, L4-S1 PLIF can achieve better sagittal balance and functional results post surgery. In contrast, L5-S1 PLIF has a higher postoperative reoperation rate. The results of our multivariate analysis revealed that, in asLDH of IS L5-S1 patients, with high-grade disc herniation and abnormal sagittal alignment, L4-S1 PLIF is more suitable during primary surgery.

## Data Availability

Data is available via a request to the corresponding author.

## Abbreviations

asLDH: Adjacent asymptomatic lumbar disc herniation
PLIF: Posterior lumbar interbody fusion
IS: Isthmic spondylolisthesis
ADS: Adjacent segment degeneration
LL: Lumbar lordosis
SL: Segmental lordosis
SS: Sacral slope
PI: Pelvic incidence
PT: Pelvic tilt,
SD: Slip degree
VAS: Visual analogue scale
ODI: Oswestry disability index
BMD: Bone mineral density
BMI: Body mass index
SFI: Subcutaneous fat index
